# Exchange of Microbiomes in Plant-Insect Herbivore Interactions

**DOI:** 10.1128/mbio.03210-22

**Published:** 2023-03-07

**Authors:** A. M. Pirttilä, V. Brusila, J. J. Koskimäki, P. R. Wäli, A. L. Ruotsalainen, M. Mutanen, A. M. Markkola

**Affiliations:** a Ecology and Genetics, University of Oulu, Oulu, Finland; b Natural Resources Institute Finland, Helsinki, Finland; Ohio State University

**Keywords:** herbivory, insect-microbe interactions, microbiome, plant-microbe interactions, symbiosis

## Abstract

Prokaryotic and eukaryotic microbial symbiotic communities span through kingdoms. The vast microbial gene pool extends the host genome and supports adaptations to changing environmental conditions. Plants are versatile hosts for the symbionts, carrying microbes on the surface, inside tissues, and even within the cells. Insects are equally abundantly colonized by microbial symbionts on the exoskeleton, in the gut, in the hemocoel, and inside the cells. The insect gut is a prolific environment, but it is selective on the microbial species that enter with food. Plants and insects are often highly dependent on each other and frequently interact. Regardless of the accumulating evidence on the microbiomes of both organisms, it remains unclear how much they exchange and modify each other’s microbiomes. In this review, we approach this question from the point of view of herbivores that feed on plants, with a special focus on the forest ecosystems. After a brief introduction to the subject, we concentrate on the plant microbiome, the overlap between plant and insect microbial communities, and how the exchange and modification of microbiomes affects the fitness of each host.

## INTRODUCTION

The current trend of climate change provokes insect expansion to new geographic areas, providing new threats, especially in the Northern Hemisphere. In North America, the impact of insect pests and pathogens of forests has been evaluated to be equal to that of fire and forestry combined (1). Invasive forest pests can eliminate keystone species and have large negative effects on biodiversity and carbon cycling, which can eventually impair ecosystem services (2). The spread of invasive species is complex and is affected by many factors, including insect lifestyle, the presence of parasitoids and predators, and the palatability level of host tissues due to, for example, phenol-richness, toxic compounds, or hard or otherwise protected plant parts (3). An important factor that is often neglected is the presence of microbial communities, both in the insect and in the plant host tissues.

Microbial symbionts of prokaryotic and eukaryotic microorganisms exist throughout kingdoms. Microbes are short-lived and possess a huge diversity of metabolic pathways, which, together with their horizontal gene transfer systems, enable rapid evolution and environmental responses (4). In symbiosis, the huge gene pool of microbial communities extends the host genome and supports adaptations to changing environmental conditions (5). Specifically, plants are prolific hosts of microbes, as they carry microbial symbionts both on the surface, called epiphytes, as well as inside tissues, known as endophytes, and even in the cells (6). The plant microbiomes consist of bacteria, fungi, oomycetes, algae, and protozoa (7). The microbial symbionts can live in virtually any plant tissue, flower, bud, seed, stem, leaf, or root (6). The mutualistic microbes of plants are typically acquired horizontally from the environment, although a vertical transmission through seeds occurs in the most intimate symbioses (5). For example, endophytic fungi are classified into four different classes, according to their transmission, biodiversity, and host range (8). A study on a conifer, namely, the Norway spruce, showed that a tree individual can host 86 to 110 fungal needle endophytes (9), whereas a single needle can be colonized by 34 different fungal species (10).

Similar to plants, insects are colonized by microbial symbionts, with their habitats being the exoskeleton, the gut, the hemocoel, and the cell interior. The insect microbiota consist of eukaryota, such as fungi and protists, as well as archaea and bacteria, and bacteriocyte symbioses are formed by intracellular microbes (11, 12). Insect microbiomes can be divided into open associations, which are invaded by external microbes, and closed associations, in which the invasion is prevented. For example, the exoskeleton and gut are open for invaders. As many as 1,000 culturable bacteria are found on the exoskeleton of the fruit fly (13). The insect gut is a prolific environment but is selective on the microbes that enter with food. The hindgut and the ileum are typically the regions with the highest counts of microbes (11). The compositions of microbial gut communities can also change along the insect development. In the gut of the African cotton leafworm (Spodoptera littoralis [Boisduval]), which is among the most detrimental agricultural pests worldwide, bacterial species belonging to *Enterococcus*, *Pantoea*, and *Citrobacter* are abundant in early life, and Clostridia becomes the most common class in adults (14).

Some insect species have cuticular structures, which are specialized in providing a niche for the microbial counterparts. Examples are the mycangia host fungi or bacteria that are needed for the insect’s offspring (11). Among insects, intracellular microbes are common in specific groups, where they are found in cells specialized for this purpose, called bacteriocytes or mycetocytes. This is a closed association, in which the endosymbionts dominating the bacteriocyte or mycetocyte, which are considered to be primary symbionts, have no access to the environment (11). In the plant sap-feeding insects, the endosymbionts are important in providing essential amino acids for their hosts, as the phloem sap is a poor nitrogen source (15). However, it is typical for this insect group to also host secondary symbionts that are associated with the bacteriocytes or mycetocytes. The secondary symbionts are similarly vertically transmitted, but they can also transfer horizontally and reside in the insect sheath cells or in the hemolymph. The secondary symbionts can provide the host with traits, such as thermal tolerance or resistance to parasitoids or fungi (11).

The two types of higher organisms, namely, plants and insects, are often highly dependent on each other and frequently interact. Regardless of the accumulating evidence on the microbiomes of both organisms, it largely remains unclear how much their microbiomes overlap and how much they modify each other’s microbiomes. In this review, we approach this question from the point of view of herbivores feeding on plants, with a special focus on the forest ecosystems. We will first take an overview on the plant-herbivore interaction and how the plant microbiome is affected by herbivory. Then, we will focus on the existing knowledge regarding the overlap between plant and herbivore microbial communities (excluding viruses). Finally, we will discuss how the modification of these microbiomes affects the fitness of each host and how they could potentially be manipulated to improve plant fitness.

## PLANT DEFENSE IN HERBIVORE INTERACTIONS

Plants have developed mechanisms to sense the presence and the type of an invader. The recognition of the invader triggers signaling pathways that lead to specific responses. A number of signaling compounds and crosstalk between the pathways are required for the activation of the defense responses, which are briefly discussed below to allow for an understanding of the intricacy of the tritrophic interactions between plants, insect herbivores, and microbes (for further details, see [16, 17]).

There are two main mechanisms to identify the invaders: pattern recognition and effector molecule-based recognition. Pattern recognition is based on elicitors and pattern recognition receptors (PRRs) that can detect a range of molecular patterns. In the case of herbivory, such patterns are called herbivorous insect-associated molecular patterns (HAMP) (17). In more detail, damage-associated molecular patterns (DAMP) are endogenous elicitors that are released, for example, from the cell wall (18). Microbe-associated molecular patterns (MAMP) or pathogen-associated molecular patterns (PAMP) are derived from microbial molecules. The recognition of the insect may also be based on the MAMP-patterns of insect endosymbionts (19). The pattern recognition initiates the PAMP-triggered immunity (PTI) (16). Besides the molecular patterns, the plant recognizes the effector molecules of pathogens by their resistance (R)-gene products, and such gene-for-gene-recognition triggers the effector-triggered immunity (ETI). For example, the components of insect saliva can serve as effectors. Pattern-based recognition occurs at the cell membrane, and the effector-based recognition is intracellular (18).

The sensing of an invader often leads to the activation of a defense pathway. The salicylic acid (SA) signaling pathway is typically triggered by biotrophic pathogens, and the jasmonic acid and ethylene (JA/ET)-mediated signaling pathway is triggered by wounds, herbivore insects, and necrotrophic pathogens. The activation of local defense responses through SA often triggers systemic acquired resistance (SAR), which requires long-distance signaling and primes the undamaged tissues (16). The association of the plant with mutualistic microbes can trigger induced systemic resistance (ISR), which also leads to priming (16, 20). Priming is an enhanced ability to respond faster and stronger to pathogen attacks (21). Basically, priming makes plants more sensitive to JA and ET, which control ISR (16). A sucking insect may also promote the plant defense against other herbivores, as the honeydew secretions of the rice brown planthopper (Nilaparvata lugens) contain members of the gut microbiome that induce systemic resistance on the monocot host, namely, rice (Oryza sativa). This further leads to the accumulation of phytoalexins and the release of volatile organic compounds that attract herbivore enemies on rice leaves (22).

The recognition of suitable and high-quality plants for feeding and reproduction is vital for herbivore insects (23), as most of them attack specific groups of plant species, with only 10% being generalists (24). Herbivorous insects can be classified in several ways, for example, based on the host taxonomy, or based on the type of consumed tissue, into algivorous (algae), frugivorous (flowers), xylophagous (wood), folivorous (leaves), granivorous (seeds), and mucivorous (sap) species, or, based on their feeding mode, into internal, external, or, alternatively, into sucking or chewing insects (25). Further, herbivores can be classified by the level of host species specialization as monophagous, oligophagous, or polyphagous feeders (i.e., those feeding on a single plant species, those that feed on several closely related species or genera, and those that feed on a wide variety of different plant groups, respectively) (26). Plants can recognize the insect species and adjust their defense responses accordingly. Overall, herbivory can trigger a cascade of community-wide interactions (27).

The defense strategy in many plants is to accumulate high quantities of compounds that are toxic to insects, whereas other plant species prefer to use their resources to minimize herbivore damage via rapid growth and development, dispersion, or choice of habitat. The selection of strategy may vary between plant genotypes from partitioning resources toward growth or defense (28, 29). Plant defense against herbivores can be divided into constitutive or induced reactions. The induced defense is often similar between plants, but the constitutive defense can change between plant species (30). The induction of the defense leads to the production of JA, systemin, oligogalacturonic acid, proteinase inhibitors, and hydrogen peroxide. The induced defense responses lack the capacity to develop a full resistance, but they can reduce the growth and survival of the insect herbivore (31). The plant defense reactions can also result in the development of physical barriers, such as lignification or resin production. Furthermore, plants typically produce volatile compounds that deter further herbivores and communicate the defense to neighboring plants (17, 30).

The range of plant defense compounds is broad with mechanisms of insect membrane disruption, suppression of metabolism, interference with signal transduction, and interference with development through imbalanced hormone regulation (30, 32). Plant defense responses may impact the settling, feeding, oviposition, fecundity, and fertility of insect herbivores (32). Herbivores attempt to overcome the plant defenses via constitutive or induced mechanisms (30), many of which are based on symbiotic microorganisms (17, 33, 34), which are discussed below. Insects that have a limited host range can lean on constitutive adaptations to survive unfavorable plant compounds, whereas herbivores with a wide range of plant species more readily use induced adaptations to overcome plant defenses. In natural ecosystems, the majority of plant-herbivore interactions reach a stalemate, in which both partners suffer but survive (30).

## PLANT-ASSOCIATED MICROBES

Even if the members of the plant microbiome are not directly associated with insects, they can play a significant role in this interaction. The microbiome can influence the nutrient status of the plant, interfere with plant-pathogen interactions, and modify the tolerance of the plant to abiotic stresses (5). On the other hand, plant pathogens and various abiotic factors can affect the plant-microbe interactions (17, 20, 35–37).

A great part of microbial interactions occurs in the plant roots, where the soil is typically the source of the microbes. A rhizobial symbiosis is formed in legumes when the plant host develops root nodules to allow for the bacterial fixation of atmospheric nitrogen (38). Rhizobia can play a role in the herbivory tolerance of legumes by inducing JA production and the development of systemic resistance (39). Another nitrogen-fixing symbiosis, called actinorhiza, is formed between the soil bacteria belonging to the genus *Frankia* and many plant species, including forest trees (40). In the red alder (Alnus rubra Bong), symbiosis with *Frankia* increased the herbivory of young leaves by the black slug (Arion rufus L.) (41). However, the accumulating evidence on actinorhizal symbiosis suggests that these symbionts can induce plant resistance, similar to rhizobia (42). There are also numerous soil bacteria living in the plant rhizosphere, which are called plant growth-promoting rhizobacteria (PGPR). The PGPR can fix nitrogen, increase the availability of nutrients, affect root growth and morphology, and promote the formation of other symbioses, such as rhizobial or mycorrhizal symbioses (43). Specifically, PGPR can induce systemic resistance of the host plant and help deter herbivores. For example, in cotton, the application of PGPR induces systemic resistance and the higher production of gossypol, which is a secondary metabolite. These responses reduced the larval feeding and development of beet armyworm (Spodoptera exigua Hübner) in PGPR-treated cotton plants (44). The inoculation of PGPR can also lead to the induced production of plant volatiles, which attract predatory earwigs (Dermaptera) toward the herbivore attack (45). However, the protection of the host plant is highly dependent on the combination of the PGPR and the herbivore species (46).

The most well-known fungal mutualists of plants, which are mycorrhiza that also dwell in the roots and rhizosphere, have been reported to influence plant herbivore status (47). Of all mycorrhizal types, arbuscular mycorrhiza (AM) is the most common, occurring in 74% of Angiosperm species. Orchid mycorrhiza is found in 9% of Angiosperm species, with ectomycorrhiza occurring in 2% of Angiosperm species, and the rarest mycorrhizal type, namely, ericoid mycorrhiza, occurring in only 1% of Angiosperm species (48). However, there are exceptions, as in the boreal forests, the dominant vegetation consists of ectomycorrhizal trees and ericoid mycorrhizal dwarf shrubs. The various mycorrhizal types, specifically arbuscular mycorrhiza, provide phosphate for the plant host, which delivers carbon in different forms to the fungus (49). The mycorrhizal status of the plant can affect the herbivore interaction. For example, mycorrhizal plants can carry more herbivore insects, but the herbivore survival is lower, meaning that the host plant therefore suffers less damage than do nonmycorrhizal plants (47). The result of the interaction also depends on the type of the herbivore, as phloem feeders, monophagous chewers, and oligophagous chewers thrive on mycorrhizal plants, but polyphagous chewers and mesophyll feeders are more successful on a nonmycorrhizal host. Of the various mycorrhizal types, AM fungi increase the performance of sucking insects, but the AM species Rhizophagus irregularis reduces chewing insect damage on the host plant more than the other mycorrhizal fungi (47). The AM fungi can induce host defense against herbivory, depending on the species of the interacting partners. For example, the AM fungi induce the defense of beggarticks (Bidens frondosa L.) to overcome cotton leafworm (S. littoralis) feeding, but they have no such effect on the cabbage moth (Mamestra brassicae L.) (50). On the other hand, the AM fungi can also suppress defenses against herbivores (51).

Besides the most intimate and specialized mycorrhizal symbiosis, endophytes that reside in all plant tissues form interactions with plants, ranging from mutualism to parasitism (6, 52). Dark septate endophytic (DSE) fungi commonly occur in plant roots, but their ecological functions are currently unclear (53). Both bacterial and fungal endophytes living in plant shoots can protect the host from environmental stresses, induce plant resistance, produce secondary metabolites to repel pathogens and herbivores, and promote plant growth (6). For example, the bacterial endophytes colonizing the shoot tips of mountain birch (Betula pubescens subsp. *czerepanovii* [N. I. Orlova] Hämet-Ahti) may promote tree recovery through sprouting after an attack by the winter moth (Operophtera brumata L.) (54). Endophytes have an important and versatile role in plant-herbivore interactions (55, 56), as they participate in the microbiome interactions and exchange, which is discussed in more detail in the sections below.

## INTERACTIONS BETWEEN THE MICROBIOMES OF PLANTS AND HERBIVORES

### Endophytes.

Many fungal endophytes can produce compounds that are toxic to herbivores (57–60). Sometimes, the toxic effects can be passed on to the second generation of herbivores, regardless of whether the herbivore is feeding on the plant that carries the endophyte (61), or even to a parasitoid, hyperparasitoid, or predator of the herbivore (62–64). In forest trees, the interaction is complicated, as tree leaves are colonized horizontally by fungal endophytes that make highly localized infections (6).

In conifers, the endophytic fungi have often been associated with the capacity to repel insect herbivores (65). As early as 1978, Carroll and Carroll (66) suggested that the foliar endophytes of conifers could be mutualistic symbionts. According to their studies on the Douglas fir (Pseudotsuga menziesii [Mirb.] Franco), the needle endophyte Rhabdocline parkeri produced a tolerance to the gall-forming needle pest Contarinia pseudotsugae (Condrashoff). They showed higher rates of mortality of the larvae in the galls of endophyte-infested needles (67). In further studies, a fraction of the metabolites from R. parkeri exhibited cytotoxicity by reducing the growth of spruce budworm (Choristoneura fumiferana Clemens) larvae (68). In more recent studies, the toxin rugulosin, which is produced by the fungal endophyte Phialocephala scopiformis, significantly reduced feeding by the spruce budworm on white spruce needles (69–72). Similarly, insecticides have been isolated and characterized from the endophytic fungi of red spruce needles (73). In another study on the Scots pine (Pinus sylvestris L.), Saikkonen et al. (74) showed that 5-year-old trees hosting endophytic fungi had reduced performance in 40% of the needles of the pine sawfly (Neodiprion sertifer Geoffroy). They found the relative growth rate of the larvae to be lower and the larval period to be longer in trees that had higher mean endophyte abundances. However, they saw no similar effects on the performance of another herbivore, namely, the aphid Scizolachnus pineti (Fabricius) (74).

On the other hand, when the life-history performances of the winter moth, birch leaf rolling weevil (Deporaus betulae L.), and birch arge (Arge clavicornis Fabricius) were studied with respect to endophyte infection in the deciduous tree mountain birch, Saikkonen et al. (74) found no correlation between herbivory and the presence of endophytes. They suggested that there is such seasonal and spatial variation in the colonization of tree leaves by endophytic fungi, affected by the neighboring vegetation, density of trees, weather, and topography of the ground, that consistent and effective defense against insects is not formed. Ahlholm et al. (75) further analyzed the presence of the two most common endophyte genera, namely, *Fusicladium* and *Melanconium*, along with the pathogenic rust fungus Melampsoridium botulinum, in mountain birch at the end of the growing season. The successes of the autumnal moth (Epirrita autumnata L.), the leaf chewer sawfly (Cladius compressicornis Lepeletier), the leaf skeletonizer sawfly (Dineura virididorsata Schmidt and Walter), and the beetle Deporaus betulae L. were dependent on the fungal densities on the mountain birch leaves only in specific cases. The sawfly D. virididorsata was more abundant with increased fungal endophyte densities and less frequent with the presence of the rust fungus M. botulinum. Similarly, the rust fungus decreased the performance of the autumnal moth on mountain birch. They suggested that fungal plant pathogens that cause premature senescence and abscission negatively affected the herbivore species that attack mountain birch just before leaf fall (75). The more consistent efficiency of endophytic fungi against insect herbivores in conifers could be explained by the fact that conifers carry needles for several years, compared to deciduous trees, which grow new leaves every year. Therefore, the interactions of endophytic fungi with conifer needles to repel insect herbivores may have developed toward higher mutualism (65).

### Microbial transmission (insects as vectors).

Many plant-associated microbes, especially pathogens, can use insects as vectors and are partially or totally dependent on insects for transmission. The transmitted microbes include fungi, bacteria, phytoplasmas, and protozoa (76). The vector-pathogen interaction is often specific, and the vector-borne plant pathogens can be transmitted externally or internally within the insect (76). For example, the insect can become smeared with bacteria or fungal spores (77).

Nonpersistent plant pathogens, also known as stylet-borne pathogens, are acquired and inoculated after short feeding periods, as some pathogens can live in the insect vector for only a few hours. Plant pathogens that thrive in the vector from one to four days are called semipersistent pathogens. In turn, persistent or “circulative” pathogens are accumulated internally and are released to a new plant through the insect mouthpart. Some may even multiply in the vector, and such propagative pathogens may live in the insect for the rest of their lives. In some cases, there is an incubation period between the acquisition and the transition of the plant pathogen (76), and sexual transmission between the vectors has also been observed (78). Although the plant pathogens vectoring in insects are best studied, it has been shown in grapevine (Vitis vinifera L.) that a phloem-feeding insect (American sap-feeding leafhopper Scaphoideus titanus Ball) can transfer full endophytic bacterial communities between plants. The transferred communities were most similar to those found in plant roots, even though *S. titanus* feeds on the stems of grapevines (56). This is not surprising, as a number of reports show plant root-colonizing bacteria, namely, PGPR, being transmitted to new plants via insects (79, 80).

An example of insect-mediated microbial transmission in a forest setting is the case of bark beetles (Coleoptera: Curculionidae: Scolytinae) and their fungal symbionts. The beetles feed and reproduce inside conifer bark and transfer a vast number of fungal symbionts between trees (81). Many of these bark beetle species live only in dead or decaying coniferous trees; however, there is a group that invades and kills healthy trees (82). The majority of fungal taxa vectoring between bark beetles and trees belong in the order Ophiostomatales (Ascomycota) (83). The fungal spores are transferred in the exoskeletons of the bark beetles, in mites vectored by the beetles, or in mycangia from one tree to another (77). These fungi can be pathogenic, parasitic, or commensalistic in both of the hosts (84). They may provide nutrition for the larvae of the bark beetle (85) and may help the beetles overcome tree defenses (86). For example, the fungus Ceratocystis polonica that vectors in the European spruce bark beetle (Ips typographus L.), helps the beetle invade the Norway spruce (Picea abies L.) via the degradation of plant defense compounds, namely, stilbenes (87). The Ophiostomatalean fungi can also produce volatiles that affect the behavior of the beetles, functioning as attractants or repellents, but the beetle behavior can vary by the fungal species (88), the developmental stage of the beetle, and the genotype of the host tree (89).

### Overlap between plant and insect microbiomes.

An insect or a plant host can maintain a multitude of different microbial symbionts, with each having specific functions in the host (6, 90). A microbial strain can be pathogenic in a plant host and beneficial for the insect host, whereas another microbe with positive effects in the plant host can be pathogenic to the insect (91, 92, Table 1). For example, Beauveria bassiana is an endophytic fungus on a wide array of plants and infects more than 700 insect species as an entomopathogen (93). In addition, the endophytes Clonostachys rosea, Metarhizium anisopliae, and Cordyceps fumosorosea of English oak (Quercus robur L.), Metarhizium anisopliae of Chinese yew (Taxus chinensis [Rehder and E.H. Wilson] Rehder), Cordyceps farinosa of European beech (Fagus sylvatica L.), and Ophiocordyceps sobolifera of cacao (Theobroma cacao L.) can act as entomopathogens (94).

**TABLE 1:** Microbiome exchange in plant-herbivore interactions^*a*^

Ecosystem	Plant species	Insect species/Feeding mode	Microbial species	Reference(s)
Forest	Douglas fir (*Pseudotsuga menziesii*)	Needle midge (*Contarinia pseudotsugae*)/Internal (gall-making), needle chewing	Endophytic fungus (*Rhabdocline parkeri*)	66 – 68
		Spruce budworm (Choristoneura fumiferana)/External (spun needles), needle chewing		
Forest	White spruce	Spruce budworm (Choristoneura fumiferana)/External (spun needles), chewing	Endophytic fungus (*Phialocephala scopiformis*)	69 – 72
Forest	Scots pine (*Pinus sylvestris* L.)	Pine sawfly (*Neodiprion sertifer*)/External (needles), chewing	Endophytic fungi	74
Forest	Lodgepole pine (*Pinus ponderosa*)	Mountain pine beetle (*Dendroctonus ponderosae*)/Internal (phloem), chewing	Phloem endophyte (Bacillus pumilus)	128
Agriculture, Forest	Many plant species	>700 insect species	Endophytic fungus (Beauveria bassiana)	93
Agriculture, Forest	Many plant species	Several insect species	Endophytic bacterium (Bacillus thuringiensis)	91
Agriculture	Grapevine (Vitis vinifera L.)	Leafhopper (*Scaphoideus titanus*)/External, sucking (sap)	Endophytic bacteria	56
Forest	Ash (*Fraxinus* spp.)	Emerald ash borer (*Agrilus planipennis*)/Internal (phloem, wood), chewing	Fungal and bacterial endophytes	100
Forest	Arizona pine (*Pinus arizonica*), Durango pine (*Pinus durangensis*)	Bark beetle (*Dendroctonus rhizophagus*)/Internal (phloem), chewing	Endophytic bacteria	101
Agriculture	Strawberry (*Fragaria ananassa*)	Caterpillar (*Vanessa cardui*)/External (leaves), chewing	Epiphytic and endophytic bacteria and fungi	106
Forest	Conifers (several species)	Bark beetles (Coleoptera: Curculionidae: Scolytinae)/Internal (phloem), chewing	Insect symbiotic fungi (Ophiostomatales)	82
Agriculture	Bittercress (*Cardamine hirsute*)	Leaf-mining fly (*Scaptomyza nigrita*)/Internal (leaf mining), chewing	Epiphytic and endophytic bacteria	103
Forest	*Pinaceae*	Red turpentine beetle (*Dendroctonus valens*)/Internal (phloem), chewing	Insect symbiotic fungi	81
Forest	Mountain pine (*Pinus contorta, Pinus contorta-Pinus banksiana*)	Mountain pine beetle (*Dendroctonus ponderosae*)/Internal (phloem), chewing	Insect symbiotic bacteria (Pseudomonas and *Rahnella*)	33
Forest	Norway spruce (*Picea abies*)	European spruce bark beetle (*Ips typographus*)/Internal (phloem), chewing	Insect symbiotic fungus (*Ceratocystis polonica*)	87

a Light grey, positive, dark grey, negative; white, neutral effect on host.

On the other hand, the plant-based diet of the insect, which consists of plant material and endophytic microbes, shapes the microbial composition of the insect gut (95–98), and this can be linked with the ability of the insect to defeat plant defenses (99, 100). In the study by Lòpez-Fernàndez et al. (56) on grapevines and the phloem-feeding grasshopper (*S. titanus*), the plant endophytic and insect gut communities were similar, but the plant communities were dominated by Proteobacteria, whereas the insect communities were dominated by Firmicutes (56). Similarly, in the emerald ash borer (Agrilus planipennis Fairmaire), which is an herbivorous pest of ash trees, the composition of the leaf microbiome was a strong predictor of the gut microbial community structure in the adult insects. The leaf microbiome of the tree explained 53% and 48% of the variation in the fungal and bacterial communities of the emerald ash borer, respectively (100). However, the bacterial endophytic communities of the roots, phloem, and bark of the pine species Pinus arizonica Engelm. and P. durangensis Martínez were significantly different from the gut communities of a bark beetle (Dendroctonus rhizophagus Thomas and Bright) that colonizes the saplings of the pines (101). Similarly, nine polyphagous caterpillar species feeding on five various tree species had clearly different gut microbiomes, compared to the host leaves, with respect to bacterial species. However, there were similarities between the fungal microbiomes of the insect guts and the host leaves (102). Therefore, the extent of the overlap between the plant and insect microbiomes is likely highly dependent on the combinations of species.

The plant microbiome is affected by insect herbivory, as well. In bittercress (Cardamine hirsuta L.), herbivory by a leaf-mining fly (Scaptomyza nigrita Latreille) led to a higher abundance of various microbiome taxa. However, the increased abundance reflected community-wide compositional shifts toward lower ecological diversity in the bittercress. Such shifts included plant-pathogenic members of the microbiome, such as Pseudomonas syringae, growing toward higher populations (103). A similar observation was made when the effect of pea aphid (Acyrthosiphon pisum Harris) was studied on the distribution of P. syringae among epiphytic communities, as the bacterium thrived better on aphid-infested leaves. The aphid feeding, attributed to the presence of honeydew, had a pronounced effect on P. syringae populations, which were initially small on the leaves (104). In beech and oak, the feeding of lepidopterous larvae caused changes in the bacterial communities of the leaves, which were associated with the increase of available nitrogen (105). However, in strawberry, the flower microbiome was primarily shaped by the plant genotype and not by Vanessa cardui L. caterpillars. Specifically, the plant volatiles, such as terpenoids and benzenoids, were mainly responsible for determining the structure of the bacterial and fungal communities of the strawberry flowers (106). This suggests that the plant microbiome is reasonably resilient to changes by herbivory or other plant-insect interactions.

### Benefits and disadvantages of obtaining new microbiome members.

In general, microbes can have a prominent effect on the host range of insects (107). For example, the red turpentine beetle (D. valens LeConte) acts as a vector for fungi that have enabled their invasion from Northern America to China (81, 108). In its native range, the beetle is only a minor threat to pine trees, but it has caused serious mortality in pines on the other continent (108). Taerum et al. (81) identified a large shift in the assemblage of the invasive red turpentine beetle fungal symbionts. They concluded that the fungal community shift had enabled the beetle to become invasive in China (81).

Adaptation to a new host can happen fast via horizontal gene transfer from microbes to insects (109), in which the insect microbiome can enable rapid adaptation to disturbances in the environment (110). Microbes may detoxify plant defense compounds and provide enzymes with which to digest the plant material (111). For example, the mountain pine beetle (D. ponderosae Hopkins) can consume terpenes with the help of gut microbes that belong to the genera Pseudomonas and *Rahnella*, which have the capacity to degrade terpenes (33). The gut microbes of wood-boring Cerambycidae, Curculionidae, and Siricidae, as well as some lepidopteran species, can produce cellulases for the food digestion of their hosts (112–115). Similarly, proteinase inhibitors that are produced by the plant host may be overcome via the hyperproduction of proteases by the symbiotic bacteria (116). In Cerambycidae, the midgut microbes also synthesize essential nutrients for the host (117–119), and they additionally possess the capacity for nitrogen fixation (120). Specifically, in the sap-feeding Hemiptera, in the process of evolution, the insects have become dependent on the biosynthesis of amino acids and vitamins by the gut microbiome, which is obtained from the host plant (11).

Mutualists of herbivorous insects that are plant pathogens can also help in the invasion of the plant by circumventing plant defenses. Such relationship may have evolved from a symbiotic association with the host, plant, or insect that was followed by adaptation to the new host (92, 121). An insect-associated microbe can suppress the plant defense via various mechanisms of manipulating plant signaling (17, 122). The weakened plant defenses can also result from a synergy between multiple microorganisms (123). For example, many phytopathogenic bacteria and fungi manipulate cytokinin signaling to attack the plant. Microbes can either produce cytokinins or modify cytokinins that are produced by the plant (124). The plant defense system can further be manipulated via the exploitation of the existing antagonism between the SA and JA signaling networks (125). Microbes can also cause indirect changes in the plant, such as altered volatile production and visual cues (17, 23, 126).

The timing of the interactions between plants, insects, and microbial symbionts may be crucial for a successful microbiome exchange. For example, in Silver birch (Betula pendula L.), the phenological synchrony between the emergence of the overwintering herbivore gypsy moth (Lymantria dispar L.) and the budding of the host plant affected herbivore survival through the gut microbiome of the insect. There was clearly a lower diversity of bacteria in the guts of asynchronous larvae than in those of synchronous larvae, which lead to the lower susceptibility of asynchronous larvae toward the entomopathogenic bacterium Bacillus thuringiensis (127).

Besides defeating plant defenses and supporting exploitation by the insect herbivore, the transmitted microbes can alter the survival, fecundity, and immunity of the insect host and can therefore alter the fitness of the herbivore (126). The manipulation of the insect microbiome can shorten the life span, increase the latent period, block pathogen transmission, change reproduction, and increase insect susceptibilities to natural enemies (90). The newly acquired microbes can cause direct and indirect changes in insect behavior, such as altered host preferences or feeding (126). For example, in the lodgepole pine (Pinus ponderosa Douglas ex C. Lawson), the beneficial phloem endophyte Bacillus pumilus is antagonistic toward a fungal symbiont of the mountain pine beetle (Dendroctonus ponderosae) (128).

Besides the plant pathogens vectoring in insects, the consequences of microbiome exchange have rarely been studied in plants. The best example is the widely studied and used B. thuringiensis, which causes toxicity to the larvae of many insects through the formation of crystal proteins but acts as a beneficial endophyte inside plants (91). When the phloem-feeding grasshopper *S. titanus* transferred an entire endophytic community to new grapevine plants, the receiver plants were healthy. Lòpez-Fernàndez et al. (56) suggested that the endophytes had a mutualistic role in the grapevine, as their earlier studies had shown that the endophytes stimulate growth and protect the grapevines from pathogens (129).

## CONCLUSIONS

**FIG 1:**
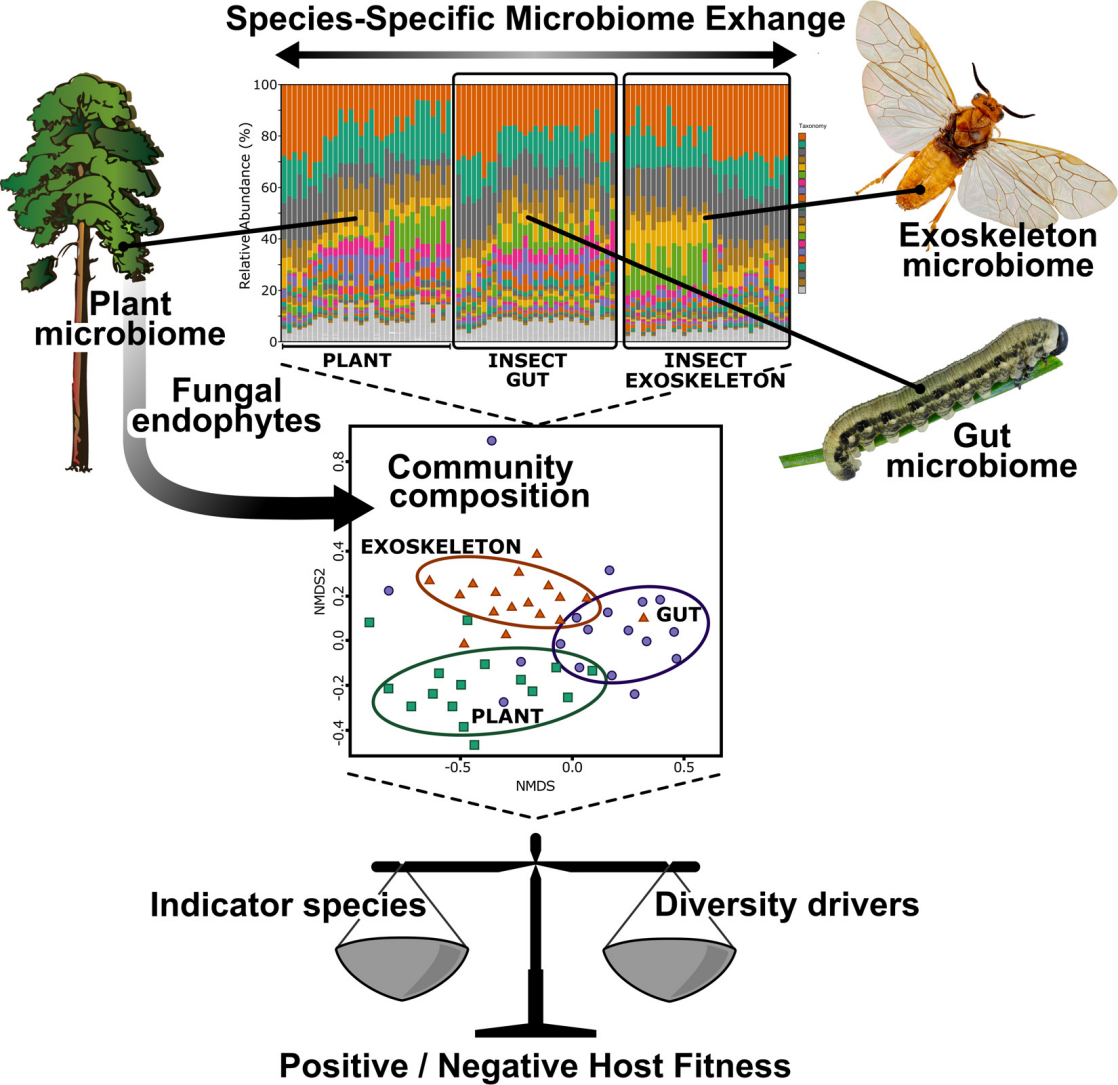
Microbiome exchange and the interactions between microbial communities of plants and insect herbivores. The microbial interaction between each species of plant and insect is likely highly specific and dependent on the insect’s feeding mode and environmental conditions. Endophytic fungi may play an important role by producing secondary metabolites that are toxic to the insect herbivores. However, together with endophytic bacteria, they can also become part of the insect gut microbiome. Experiments revealing the microbial community structures will enable the identification of the drivers of diversity and the indicator strains for increasing or reducing the fitness of each host as well as the further manipulation of the microbiome exchange.

Gupta and Nair (130) recently summed up the various roles of gut microbes in the survival and adaptation of insects to specific environments. However, no great generalizations can be made regarding the interactions between plant and herbivore microbiomes or on how the hosts affect each other’s microbial communities and further fitness. The interactions between each species of plant, each species of insect, and their microbiomes is likely highly specific and dependent on the feeding mode and environmental conditions of the insect (Fig. 1; Table 1). As data from high-throughput sequencing studies accumulate, it becomes more evident that the microbial transfer between plants and insects is much more extensive than previously thought. Therefore, studies similar to the ones performed by Lòpez-Fernàndez et al. (56) on the bacterial communities in the sap-feeding grasshopper and grapevine, by Mogouong et al. (100) on the microbial communities in the emerald ash borer and ash trees, by Šigut et al. (102) on different caterpillar and tree species, and by Gonzalez-Escobedo et al. (101) on the bacterial communities in the bark beetle and pine trees, should become mainstream. Such studies would provide information on the involvement of microbial symbionts in the performance of an herbivore on a specific plant species and in a given environment, such as forest ecosystems. For example, the expansion of many forest pests, such as the invasive woodwasp (Sirex noctilio Fabricius) and spruce sawfly (Gilpinia hercyniae Hartig) has thus far been explained by the lack of parasitoids in the new environment (131, 132). However, the exchange of microbial communities with new host plants may have played an important role in their success in spreading to the new areas.

Studies on the transmission of microbial communities by insects could further revolutionize approaches to plant protection and even plant growth improvement. The analysis of the microbial community structures in each host, plant and insect will enable the identification of the drivers of diversity and the indicator strains for specific conditions (Fig. 1). The next step could be manipulations of the microbiome exchange. This could take place through the enrichment of species of bacteria or fungi that are beneficial for the plant in that they enhance plant growth, kill pathogens or pests (133), or eliminate plant-pathogenic strains through, for example, phage-based strategies (134, 135). The microbial biocontrol of herbivores would provide an environmentally friendly alternative to chemical pesticides that are, to a great extent, responsible for the global insect decline (17, 135–139). Therefore, research on the interactions between the microbial communities of plants and insects remains highly important for harnessing microbiome exchanges for forest management and crop production in the future.
